# A Polygenic Score Predicts Caries Experience in Elderly Swedish Adults

**DOI:** 10.1177/00220345241232330

**Published:** 2024-04-07

**Authors:** N. Fries, S. Haworth, J.R. Shaffer, A. Esberg, K. Divaris, M.L. Marazita, I. Johansson

**Affiliations:** 1 Umeå University, Umea, Sweden; 2 University of Bristol, Bristol, UK; 3 University of Pittsburgh, Pittsburgh, PA, USA; 4 University of North Carolina at Chapel Hill, Chapel Hill, NC, USA

**Keywords:** tooth diseases, tooth demineralization, human genetics, epidemiology, risk factors, genetic risk score

## Abstract

Caries is a partially heritable disease, raising the possibility that a polygenic score (PS, a summary of an individual’s genetic propensity for disease) might be a useful tool for risk assessment. To date, PS for some diseases have shown clinical utility, although no PS for caries has been evaluated. The objective of the study was to test whether a PS for caries is associated with disease experience or increment in a cohort of Swedish adults. A genome-wide PS for caries was trained using the results of a published genome-wide association meta-analysis and constructed in an independent cohort of 15,460 Swedish adults. Electronic dental records from the Swedish Quality Registry for Caries and Periodontitis (SKaPa) were used to compute the decayed, missing, and filled tooth surfaces (DMFS) index and the number of remaining teeth. The performance of the PS was evaluated by testing the association between the PS and DMFS at a single dental examination, as well as between the PS and the rate of change in DMFS. Participants in the highest and lowest deciles of PS had a mean DMFS of 63.5 and 46.3, respectively. A regression analysis confirmed this association where a 1 standard deviation increase in PS was associated with approximately 4-unit higher DMFS (*P* < 2 × 10^−16^). Participants with the highest decile of PS also had greater change in DMFS during follow-up. Results were robust to sensitivity analysis, which adjusted for age, age squared, sex, and the first 20 genetic principal components. Mediation analysis suggested that tooth loss was a strong mediating factor in the association between PS and DMFS but also supported a direct genetic effect on caries. In this cohort, there are clinically meaningful differences in DMFS between participants with high and low PS for caries. The results highlight the potential role of genomic data in improving caries risk assessment.

## Background

Dental caries remains a major global health problem in both high- and low-income countries (World Health Organization 2022). Caries is a multifactorial disease where disease manifestation is influenced by host susceptibility and environmental, behavioral, and microbial factors (Chapple et al. 2017). Host genetic factors may, therefore, play a role in determining disease experience, and genetic effects are reported to explain around 50% of the variation in caries experience (Shaffer et al. 2015; Haworth et al. 2020). Genome-wide association studies (e.g., Morrison et al. 2016; Shungin et al. 2019; Alotaibi et al. 2021) use a systematic approach to identify the common genetic variants across the genome that influence caries risk. While these studies help provide new insight into disease biology, another major motivation for performing them is to drive novel clinical applications incorporating genomic information in health care.

One such application involves the use of polygenic scores (PSs) to perform risk stratification in a clinical setting. PSs can be constructed using various methods (Ni et al. 2021), which all aim to aggregate the effects of multiple genetic variants into a single score representing an individual’s overall genetic liability to disease. Theoretically, a PS for disease can be used as an adjunct to clinical assessment, with or without other biomarkers, to help identify patients at high risk of disease and thereby offer more tailored treatment or prevention to these individuals. PSs can effectively identify individuals at risk of various outcomes, including stroke (Hachiya et al. 2020; Li et al. 2021; Neumann et al. 2021) and type 2 diabetes (Liu et al. 2021; Ashenhurst et al. 2022; DiCorpo et al. 2022).

There is growing excitement around the clinical applications of PSs (e.g., the Our Future Health program), which will seek to generate such scores for 5 million participants in the United Kingdom (Genomics PLC, 2022). Yet there are few applications in dentistry, and it is currently unclear whether a PS would effectively estimate caries outcomes and aid in caries risk assessment.

This study aimed to test whether a PS for caries is associated with incident or prevalent caries in a cohort of elderly Swedish adults. This will help gauge the feasibility and potential value of genomic risk stratification in dentistry.

## Methods

### Ethics Statement

The project was approved by the Swedish Ethical Authority Dnr 2020-01416 and the SIMPLER steering group. All participants gave consent.

### Parent Cohort

The study included adult Swedish participants from the Swedish Infrastructure for Medical Population-Based Life-Course and Environmental Research (SIMPLER) project (https://www.simpler4health.se/about-us/). SIMPLER originated as 2 longitudinal cohorts—the Swedish Mammography Cohort and the Cohort of Swedish Men, established in 1987 and 1997, respectively (Harris et al. 2013), and are now managed under a single infrastructure. Male participants were born between 1918 and 1952, while female participants were born between 1914 and 1948. All participants were living in central Sweden at the time of recruitment. SIMPLER contains around 107,000 participants and is the parent infrastructure for several nested substudies with detailed phenotypic and biological information.

Biological samples used for genetic data generation came from 3 sources. The largest batch (termed batch 1 or SIMPLER) included ~33,000 participants who donated saliva between 2005 and 2008. The next largest batch (termed batch 2 or COSM) included ~7,500 participants in the Västerås subcohort who donated blood between 2010 and 2019. The smallest batch (termed batch 3 or SMCC) included ~5,000 participants in the Uppsala subcohort who donated blood between 2003 and 2009. Current information about the SIMPLER subcohorts can be found at https://www.simpler4health.se/researchers/cohorts/.

### Genetic Data Generation

DNA extraction, genotyping, and routine quality control of genetic data were carried out as described in Appendix Table 1.

Principal component analysis was carried out on participants passing routine quality control, with genetic principal components only reported for unrelated participants (second degree). For the present study, the analysis was restricted to participants with nonmissing principal component variables (i.e., the subset of unrelated participants; *n* = 15,460).

Genotype phasing and imputation were carried out to the Haplotype Reference Consortium imputation panel (McCarthy et al. 2016) r1.1 panel using Eagle v2.4 and minimac v4 implemented by the Michigan Imputation Server.

### Dental Data

Information on dental status was retrieved from the Swedish Quality Register for Caries and Periodontitis (SKaPa, www.skapareg.se) and matched to SIMPLER genetic data using a unique personal identification number. SKaPa contains information about the condition of each tooth surface as recorded by an examining dentist or dental hygienist in public or private dental offices. Dental examinations included a visual and tactile inspection of cleaned and dried teeth using explorers, mirrors, and bite-wing radiographs when a visual inspection was impossible. For the incisor and canine teeth, 4 surfaces were scored, and for premolar and molar teeth, 5 surfaces. Third-molar teeth were excluded. Caries lesions were classified as follows: D0 = for untreated and clinically sound tooth surfaces, D1 = caries in the outer enamel, D2 = caries extending into the enamel–dentin border, and D3 = caries in the dentine. Surfaces with a fissure sealant, enamel hypoplasia, fluorosis, or tooth wear were recorded as D0. Restored surfaces were scored as D3. For missing or crown-covered incisors and canines, 4 surfaces were scored as caries affected, and for premolar and molar teeth, 5 surfaces. Nonerupted teeth and congenitally missing teeth were imputed as caries free.

The timing of dental data acquisition depended on when a participant presented for complete dental charting, which is typically annually or every 2 y in Sweden. Data were available between 2010 and 2019. In cases where data from multiple visits were available for a participant, the first visit was used in cross-sectional analysis. Longitudinal analysis was carried out in a subset of participants with data from at least 3 visits spanning at least 2 y of follow-up. All age variables used in analysis refer to age at dental examination.

### Inclusion Criteria

The analyses included participants who 1) passed genomic data quality control measures described in Appendix Table 1 after filtering for ancestry and cryptic relatedness and 2) had valid caries data available from complete dental charting on at least 1 occasion. There were no restriction criteria for age or general health condition.

### PS Construction

Data for model training were taken from the largest available genome-wide association study for caries (Shungin et al. 2019). This is a consortium-based genome-wide meta-analysis that included analyses of both clinical caries traits and a self-reported proxy trait. Importantly, the SIMPLER cohort did not contribute to this consortium, and there is no known sample overlap between the training and test data sets in SIMPLER.

Model training was carried out in a genome-wide approach using the QuickPRS method implemented in LDAK5.2. QuickPRS is a summary statistic implementation of the LDAK-BayesR-SS method (Zhang et al. 2021), which uses a Bayesian multiple regression approach incorporating functional information when assigning weights to genetic variants in the prediction model. This method was selected as it is reported to outperform other available methods in the original methods paper (Zhang et al. 2021) and has good performance compared to other methods in an independent comparison (Ni et al. 2021). LDAK precomputed tagging files were provided by the author of the method (https://dougspeed.com/quick-prs/) and originally constructed in a subset of participants of British ancestry in the UK Biobank (Bycroft et al. 2018).

The PS included variants from chromosomes 1 to 22. After generating model weights, PSs were constructed for SIMPLER participants using the score function in PLINK2 (Chang et al. 2015). Scores were generated in each genotyping batch separately and first summarized across chromosomes 1 to 22 and then standardized to a mean of 0 and standard deviation of 1 within each batch.

### Cross-Sectional Tests for Association with Caries Traits and Number of Teeth

In the primary analysis, PSs were assigned into deciles, and caries experience was summarized in each decile as the mean (standard deviation) of decayed, missing, and filled tooth surfaces (DMFS) and mean (standard deviation) of number of teeth.

Sensitivity analyses were carried out using linear regression models in R (version 4.2.1). Unadjusted and fully adjusted models (including adjustment for age, age squared, sex, and genetic principal components) were fitted. Sensitivity analyses were stratified by genotyping batch.

Mediation models were fitted using the “mediation” package in R (Tingley et al. 2014) to evaluate whether the effects of the genetic score on DMFS are partially mediated through genetic effects on tooth loss. This analysis is conceptually similar to a sensitivity analysis of PS association with DFS. Still, it has the advantage of explicitly modeling the number of lost teeth, thereby providing greater information to the model than excluding the missing component in DMFS.

### Tests for Association with Longitudinal Change in Caries Experience

Longitudinal analysis tested for association between PS and rate of change in DMFS during follow-up. A 2-stage processes was employed. First, a random-intercept, random-slope linear mixed model was fitted using the “lme4” package in R (Bates et al. 2015), using the observed DMFS at each visit as the Y variable and participant age at each visit as the X variable. The random effects for slope in this model represent per-participant variation in the rate of change in DMFS during follow-up.

In the second stage, the random effects for slope were adjusted for baseline DMFS and then summarized as the mean (standard deviation) slope in each decile. Tests for association between PS and slope were carried out using linear regression, with adjustment as per the cross-sectional analysis.

The study is reported according to Strengthening the Reporting of Observational Studies in Epidemiology criteria, and a checklist is provided as online appendix material.

## Results

In total, 15,460 participants met the inclusion criteria and were included in the cross-sectional analyses. A subset of 9,939 participants were included in the longitudinal analysis, with a mean follow-up period of 6.5 y, corresponding to around 64,000 person-y of follow-up. The mean age of study participants at the first dental visit was 73.6 y (Table 1). Overall, the study included more male than female participants, with differences between the 3 genotyping batches reflecting that the underlying cohort studies recruited males and females separately. The population had a relatively high burden of caries experience with a comparably modest prevalence of tooth loss (Table 1).

**Table 1.** Demographic Information of the Cohort Participants Included in the Analyses.

Sample	Number in Cross-Sectional Analysis	Mean (SD) Age at First Dental Visit, y	% Female Sex	Mean (SD) Number of Teeth Present at First Dental Visit	Mean (SD) DMFS at First Dental Visit	Number Included in Longitudinal Analysis	Mean Follow-up Period, y
Batch 1 (SIMPLER)	10,455	74.1 (8.1)	19.9	23.4 (6.3)	51.6 (36.7)	6,512	6.0
Batch 2 (COSM)	2,766	71.6 (7.7)	37.6	23.8 (5.5)	59.5 (30.3)	1,973	7.6
Batch 3 (SMCC)	2,239	73.7 (7.2)	100	23.6 (5.4)	61.4 (30.2)	1,454	7.1
Overall	15,460	73.6 (7.9)	34.6	23.5 (6.1)	54.5 (35.0)	9,939	6.5

DMFS, decayed, missing, and filled tooth surfaces.

The PS included 902,205 variants. Most variants had very small weights in the prediction. The distribution of variants that contributed most to the prediction is illustrated in Appendix Figure 1, and information about the 10 variants with greatest weight is provided in Table 2.

**Table 2.** Summary of the 10 Genetic Variants Contributing Greatest Weight to the Polygenic Score.

Variant	Allele 1	Allele 2	Effect	Weight	Annotation
5:134509677	C	T	–0.021	0.018	Within *PITX1-AS1*
15:63636878	C	T	–0.015	0.014	Intronic, *CA12*
19:49206985	G	A	–0.015	0.014	Missense, *FUT2*
2:25141538	A	G	–0.012	0.012	Missense, *ADCY9*
5:134509987	C	T	–0.014	0.011	Within *PITX1-AS1*
10:77866732	T	C	–0.0091	0.0091	Intronic, *C10ORF11*
17:79365861	C	T	0.012	0.0088	Near *BAHCC1*
6:26233387	A	G	0.016	0.0087	Highly correlated with missense variation in *HIST1H2BE*
22:45728370	G	A	–0.015	0.0082	Missense, *FAM118A*
15:78894339	G	A	–0.012	0.0081	Synonymous, *CHRNA3*

Effect refers to the per-allele effect of that variant. Weight represents the overall contribution to the score, taking account of both the effect size and allele frequency.

**Figure 1.**
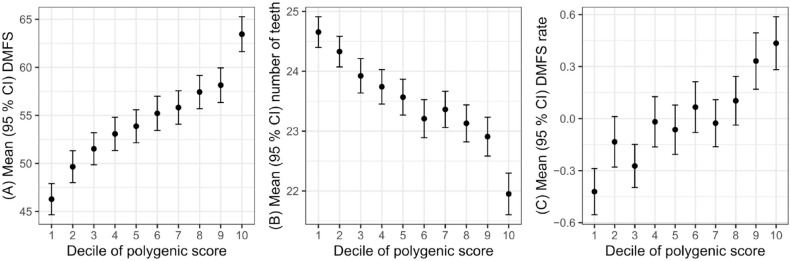
Dental caries traits by decile of polygenic score. Decayed, missing, and filled tooth surfaces (DMFS) at first visit (**A**), number of teeth present at first visit (**B**), and rate of change in DMFS (**C**) by deciles of polygenic score (PS). Deciles of the PS are ordered from 1 (lowest decile) to 10 (highest decile). The mean number of remaining teeth and DMFS scores for each decile group are shown on the y-axes. The error bars represent 95% confidence intervals for the means in each decile.

In the main cross-sectional analysis, higher PSs were associated with higher DMFS. Participants in the highest decile of PS had a mean DMFS of 63.5 compared to a mean DMFS of 46.3 for those in the lowest decile (*P* < 2 × 10^−16^) (Fig. 1A). Participants in the highest decile of PS had a mean number of teeth of 22.0 compared to a mean number of teeth of 24.7 for those in the lowest decile (*P* < 2 × 10^−16^) (Fig. 1B).

In cross-sectional regression analysis, 1 standard deviation greater PS was associated with approximately 4-unit greater DMFS. The effect estimates were consistent across the 3 batches, with minimal attenuation in effect estimates in the fully adjusted model. Greater PS was also associated with fewer teeth at the time of examination, with little attenuation in effect estimates in a fully adjusted model. The variance explained by the PS was small for both traits (i.e., in the region of 1% to 2%) (Table 3).

**Table 3.** Summary of Linear Regression Models.

Sample	*n*	Unadjusted Model			Fully Adjusted Model		
		Beta (SE)	*P*	*R* ^2a^	Beta (SE)	*P*	*R* ^2^
DMFS at first visit							
Batch 1	10,455	4.75 (0.36)	<2 × 10^–16^	0.017	4.19 (0.33)	<2 × 10^–16^	0.013
Batch 2	2,766	4.14 (0.57)	5.9 × 10^–13^	0.019	4.19 (0.57)	1.9 × 10^–13^	0.019
Batch 3	2,239	4.41 (0.63)	4.6 × 10^–12^	0.021	4.03 (0.63)	2.4 × 10^–10^	0.017
Overall	15,460	4.59 (0.28)	<2 × 10^–16^	0.017	4.18 (0.26)	<2 × 10^–16^	0.014
Number of teeth at first visit							
Batch 1	10,455	–0.80 (0.061)	<2 × 10^–16^	0.016	–0.74 (0.058)	<2 × 10^–16^	0.014
Batch 2	2,766	–0.54 (0.10)	2.8 × 10^–7^	0.010	–0.54 (0.10)	6.7 × 10^–8^	0.010
Batch 3	2,239	–0.64 (0.11)	3.0 × 10^–8^	0.014	–0.59 (0.11)	1.6 × 10^–7^	0.011
Overall	15,460	–0.73 (0.049)	<2 × 10^–16^	0.014	–0.68 (0.046)	<2 × 10^–16^	0.013
Rate of change in DMFS							
Batch 1	6,512	0.29 (0.061)	<2 × 10^–16^	0.0093	0.33 (0.031)	<2 × 10^–16^	0.012
Batch 2	1,973	0.095 (0.039)	0.014	0.0027	0.15 (0.038)	1.3 × 10^–4^	0.0060
Batch 3	1,454	0.13 (0.044)	0.0031	0.0050	0.16 (0.043)	2.7 × 10^–4^	0.0070
Overall	9,939	0.24 (0.023)	<2 × 10^–16^	0.0079	0.28 (0.023)	<2 × 10^–16^	0.011

Beta coefficients are on a tooth-surface scale for DMFS, tooth-scale for the number of teeth present, and surfaces of DMFS per year scale for rate of change in DMFS. The fully adjusted model included adjustment for age (at dental examination), age squared, sex (omitted for batch 3 with women only), and the first 20 genetic principal components.

DMFS, decayed, missing, and filled tooth surfaces.

a *R*^2^ describes the improvement in *R*^2^ by including the polygenic score in the model.

In the longitudinal analysis, the PSs were associated with a variable capturing rate of change in DMFS during follow-up, with the most rapid rate of change seen in the highest decile of PS (Fig. 1C). This association was recapitulated in regression analysis where 1 standard deviation greater PS was associated with approximately 0.2 surfaces/y higher rate of change in DMFS during follow-up (Table 3). The variance in rate of change in DMFS explained by the PS was small.

**Figure 2.**
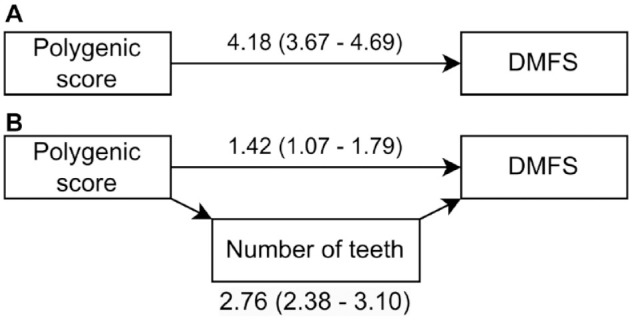
Path diagram for the mediation analysis. (**A**) The total effect in an unmediated model. (**B**) The relative importance of the direct and mediated pathways. Path weights are obtained from fully adjusted mediation models.

Given that the PS is associated with the number of teeth, we hypothesized that genetic effects on DMFS might be partially mediated by tooth loss. We tested this in a cross-sectional mediation analysis framework. The mediation analysis results supported a strong mediation pathway through the number of teeth, where approximately 66% of the total effect of the PS acted through this pathway (Fig. 2, Appendix Table 3). However, the results also supported a direct effect of PS on DMFS, which was not explained by tooth loss (Fig. 2).

## Discussion

In this study, a PS for caries was associated with a greater lifetime burden of caries in cross-sectional analysis and with greater rate of change in DMFS during follow-up, with clinically meaningful differences between participants in the highest versus lowest score deciles. This is consistent with what is reported for other complex traits, such as type 2 diabetes and cardiovascular diseases (Lambert et al. 2019), and consistent with the expectation given that caries is known to be a partially heritable disease (Shaffer et al. 2015; Haworth et al. 2020).

The participants in the present study population were around 74 y old and born in the midst of the 20th century, when free dental care for children and systematic caries prevention, such as fluoride rinsing programs, were not yet launched in Sweden. Thus, most children in Sweden in those days had a high burden of caries and/or amalgam restorations (Hugoson et al. 2000; Ordell 2012). Over time, progressing disease and tooth fracturing of amalgam-restored teeth led to extensive therapies, such as crowns, bridges, implants, and extractions leading to the high DMFS scores in the present study group. The PS for caries associated with the number of present teeth and genetic effects on tooth loss were strong mediators of the association between the PS and DMFS. This is consistent with the expectation given that caries is a cause for tooth loss and that missing teeth are counted in the DMFS caries phenotype. It is, however, possible that the PS associates with other causes of tooth loss, such as tooth loss due to periodontal disease or crown fractures, but we noted that there was also evidence for a direct effect not mediated by tooth loss. Given that the prevalence and causes of tooth loss vary by age and population, there may be value in reexamining the identified association in different populations, including those with low levels of tooth loss, and this is suggested as a topic for future research.

In this population, the PS was associated with rate of change in DMFS during follow-up, but with slightly weaker variance explained than in the cross-sectional analysis. In populations with a high burden of DMFS, there is a risk of saturation effects during follow-up, since individuals with high DMFS at the first visit have few surfaces that are suspectable to develop caries. To reduce bias from this effect, analysis of rates of change in this experiment included adjustment for DMFS at the first visit, but this provides an additional reason why it may be useful to repeat the experiment in a younger population with lower burden of DMFS.

Caries has a polygenic architecture with strong genetic correlations with a range of other diseases as well as behavioral and educational attainment (Shungin et al. 2019). This suggests the polygenic score for caries will itself be complex with a range of potential mechanisms and causal pathways creating associations between the score and caries traits in the present study group. The score is anticipated to capture variation in biological processes that predispose to caries, such as those in tooth development, innate immunity and microbial colonization, salivary flow and pH-neutralizing power (Pitts et al. 2017), and behavioral traits, such as sweet taste preference (Eriksson et al. 2019). It is therefore possible to hypothesize a wide range of underlying causal pathways, including routes that involve genetic effects on the cariogenic environment at the tooth–biofilm interface. One example is if the PS associates with food preferences, which are known to be partially heritable (May-Wilson et al. 2022) and associate with both caries incidence and supragingival microbiome (Eriksson et al. 2019). These gene-to-environment-to-disease pathways are analogous to the “nature of nurture” effect proposed for educational attainment (Kong et al. 2018). In the context of risk assessment, it is not necessary to understand the underlying causal pathways, but care will be needed to interpret the derived caries PS in other research settings, since the underlying causal pathways may be complex.

The performance of a PS is determined by the properties of the underlying training data set. While the genome-wide association study (GWAS) used for model training is the best-powered study of caries currently available, it has less power than the very large GWAS studies available for other complex traits such as anthropometric traits (Yengo et al. 2022). This is reflected in the overall variance explained by the score in the present study, which was less than that seen for other complex traits (Zhang et al. 2021). A challenge in polygenic score research is finding scores that can perform well in understudied populations (Novembre et al. 2022), but the generalizability of polygenic scores can be improved by incorporating information from genome-wide association studies in diverse populations (Graham et al. 2021). There is, therefore, a clear need to carry out further well-powered genome-wide association studies for caries in diverse populations, which is a goal of the GLIDE2 consortium (Divaris et al. 2022).

The major strengths of the present study include the large sample size in a representative, population-based sample of elderly Swedish men and women with dental data obtained from primary care clinics and the availability of longitudinal data. One limitation worth considering when interpreting the data is that, although the dental data are clinically recorded, this is done by a large number of dental personnel with no strict calibration beyond the directions given by the Swedish National Board of Health and Welfare (https://www.socialstyrelsen.se/en/). We are presently evaluating the validity of SKaPa register–derived caries data with preliminary results indicating an acceptable conformity. The large number of missing/crown-covered teeth may have led to an overestimation of caries burden in some participants in this study. However, we still expect the ranking of individuals to be acceptable and do not anticipate that routine errors in dental charting will correlate with host genotype.

This exploratory study focused on the feasibility and performance of a PS in isolation. This is unlikely to mirror a real-world clinical application since it is unrealistic to expect a PS to provide accurate information on risk by itself (Sud et al. 2023). Instead, the clinical value of a PS will likely be to supplement clinical assessments of conventional risk factors, where there are currently several caries risk assessment systems in clinical use (Cheng et al. 2022). A recent comparison of 10 caries risk assessments using clinical data found highly variable results, and currently, none of the evaluated systems was recommended for decision-making in patient care (Halasa-Rappel et al. 2019). Therefore, a combined genetic and clinical risk assessment with a conventional risk-scoring system is suggested as a topic for future research, ideally in a large cohort with longitudinal data.

## Author Contributions

N. Fries, contributed to data analysis and interpretation, drafted and critically revised the manuscript; S. Haworth, contributed to conception, design, data analysis and interpretation, drafted and critically revised the manuscript; J.R. Shaffer, A. Esberg, K. Divaris, M.L. Marazita, contributed to data interpretation, critically revised the manuscript; I. Johansson, contributed to conception, design, data acquisition and interpretation, drafted and critically revised the manuscript. All authors gave final approval and agree to be accountable for all aspects of the work.

## Supplemental Material

sj-docx-1-jdr-10.1177_00220345241232330 – Supplemental material for A Polygenic Score Predicts Caries Experience in Elderly Swedish Adults Supplemental material, sj-docx-1-jdr-10.1177_00220345241232330 for A Polygenic Score Predicts Caries Experience in Elderly Swedish Adults by N. Fries, S. Haworth, J.R. Shaffer, A. Esberg, K. Divaris, M.L. Marazita and I. Johansson in Journal of Dental Research
