# Supplementary material for: A Polygenic Score Predicts Caries Experience in Elderly Swedish Adults
Source: J Dent Res. 2024 Apr 7;103(5):502–8. doi: 10.1177/00220345241232330 (PMC11047011; doi:10.1177/00220345241232330)
Supplement: sj-docx-1-jdr-10.1177_00220345241232330 – Supplemental material for A Polygenic Score Predicts Caries Experience in Elderly Swedish Adults [file sj-docx-1-jdr-10.1177_00220345241232330.docx]

**Supplemental appendix**

**A Polygenic Score for Caries Predicts Caries Experience in Elderly Swedish Adults**

N. Fries, S. Haworth, J. R. Shaffer, A. Esberg, K. Divaris, M. L. Marazita, I. Johansson

**Appendix Table 1**. Summary of genotype data generation and quality control

| **Batch** | **SIMPLER** | **COSM** | | **SMCC** |
| --- | --- | --- | --- | --- |
| DNA source | Saliva | Blood | | Blood |
| DNA extraction | QIAamp DNA Blood Midi Kit (Qiagen) | | | Chemagen magnet bead extraction kit (Revvity Inc, Waltham, MA, USA). |
| Genotyping array | Illumina Infinium Global Screening Array (GSA-MD V3) (Illumina Inc, San Diego, California, USA) | | | Illumina Infinium Global Screening Array (GSAMD-24v1-0_20011747_A1). |
| Software for genotype calls | GenomeStudio 2.0.4 (Illumina Inc, San Diego, California, USA) | | | GenomeStudio 2.0.3 (Illumina Inc, San Diego, California, USA) |
| **Per sample quality control (exclusion criteria)** | | | | |
| Overall missingness | Missing > 2% (autosomes)  Missing > 5% (chromosome X) | | | Missing > 2% autosomes  Missing > 2% chromosome X |
| Ancestry | Non-European ancestry | | | |
| Heterozygosity outliers | +/- 3 interquartile ranges from quartile 1 / 3 | | | |
| **Per site quality control (exclusion criteria)** | | | | |
| Missingness | > 2% | | | |
| Violations of Hardy Weinberg equilibrium | HWE P < 1x10-7 | | | |
| Minor allele frequency | < 0.5% | |  | |
| Minor allele count |  | | <20 | |
| Presence in imputation panel | Variants removed which were not present in or HRC with non-matching alleles | | | |
| Allele frequency difference between reported and expected in 1000G/HRC | > 0.2 | | > 0.15 | > 0.2 |
| Duplicated variants | First copy kept | | | |
| Multi-allelic variants and insertion/ deletions | Only SNPs kept | | | |

**Acronyms**

1000G: 1000 genomes project

HRC: Haplotype reference consortium

HWE: Hardy-Weinberg equilibrium

SNP: Single Nucleotide Polymorphism

**Appendix Table 2**. Summary of path coefficients from mediation analysis. The fully adjusted model included adjustment for age (at dental examination), age squared, sex and the first 20 genetic principal components. All confidence intervals are obtained from bootstrap.

|  | **Unadjusted model** | **Fully adjusted model** |
| --- | --- | --- |
| **Path** | **Coefficient (95% CI)** | **Coefficient (95% Ci)** |
| ACME (mediated effect) | 3.07 (2.71-3.42) | 2.76 (2.38-3.10) |
| ADE (direct effect) | 1.48 (1.12-1.90) | 1.42 (1.07-1.79) |
| Total effect | 4.55 (4.03-5.08) | 4.18 (3.68-4.68) |
| Proportion of effect mediated through number of teeth | 0.67 (0.61-0.74) | 0.66 (0.60-0.73) |


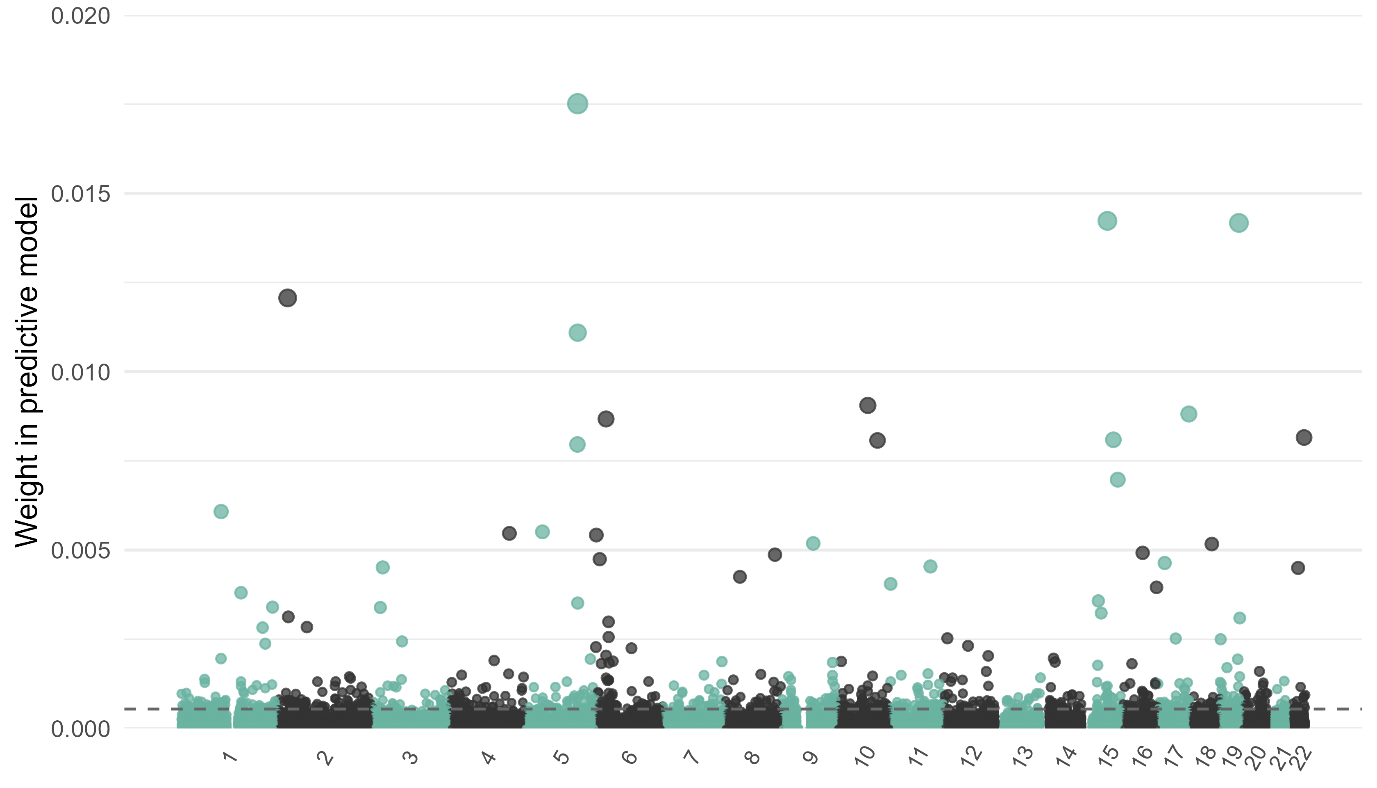


**Appendix Figure 1.** Overview of the distribution of variants contributing greatest weight to the PS. The X-axis indicates the genomic position from chromosomes 1-22. The Y-axis indicates weight in the predictive model. The dashed line on the Y-axis indicates the 99.9^th^ percentile of weight, i.e., only the 0.1% of variants with the largest weight in the score appear above the dashed line.
